# Emotional Dysregulation as a Mediator Between Childhood Adversity and Negative Urgency in Borderline Personality Disorder

**DOI:** 10.7759/cureus.102953

**Published:** 2026-02-04

**Authors:** Poojitha Konda Reddy, Kancharla Suresh Reddy, Gunde Surekha, Madhu Vamsi Ganduri, Kacham R Mohana, Azra Fatima, Nidhi R, Khande P Kumar, Amulya Arremsetty, Akhileshwar V Reddy

**Affiliations:** 1 Psychiatry, Ayaan Institute of Medical Sciences, Hyderabad, IND; 2 Psychiatry, Malla Reddy Medical College for Women, Hyderabad, IND; 3 Psychiatry, MV Health, Hyderabad, IND; 4 Psychiatry, Government Medical College and Hospital, Narayanpet, IND; 5 Community Medicine, Nims University, Jaipur, IND

**Keywords:** biosocial theory, borderline personality disorder (bpd), childhood adversity, dialectical behavior therapy (dbt), emotional dysregulation, emotion regulation, impulsivity, indian clinical sample, mediation analysis, negative urgency

## Abstract

Background: The biosocial theory of borderline personality disorder (BPD) posits that emotional dysregulation develops from the transaction between a biological vulnerability and an invalidating environment (e.g., childhood adversity), and in turn leads to behavioral dyscontrol. This study aimed to empirically test this core mechanistic pathway in a non-Western clinical sample.

Methods: We hypothesized that the relationship between childhood adversity and the hallmark BPD trait of negative urgency (acting impulsively when distressed) would be mediated by difficulties in emotion regulation. A cross-sectional study was conducted with 45 adult patients with a Diagnostic and Statistical Manual of Mental Disorders, 5th Edition (DSM-5) diagnosis of BPD in Hyderabad, India. Participants completed the Childhood Experience of Care and Abuse Questionnaire (CECA-Q), the Difficulties in Emotion Regulation Scale-Short Form (DERS-SF), and the Short UPPS-P (urgency, premeditation, perseverance, sensation seeking, and positive urgency) Impulsive Behaviour Scale. A bootstrapping-based mediation analysis (Hayes’ PROCESS Model 4) was used to test the hypothesized indirect effect.

Results: The analysis revealed a significant indirect effect of childhood adversity on negative urgency through emotional dysregulation (Indirect Effect = 0.31, 95% bootstrapped CI: 0.14, 0.52). The direct effect of childhood adversity on negative urgency, which was significant in the initial correlation, became non-significant (B = 0.08, p = 0.450) after accounting for the mediator. This pattern is consistent with full mediation.

Conclusion: This study provides strong, mechanism-based support for the biosocial model of BPD in an under-researched Indian context. The findings demonstrate that the pathogenic impact of childhood adversity on emotion-based impulsivity is explained by its disruption of emotion regulation capacities. This offers a clear empirical rationale for prioritizing emotion regulation-focused psychotherapies, such as dialectical behavior therapy, as the primary treatment approach for BPD.

## Introduction

The biosocial theory of BPD

The etiology of borderline personality disorder (BPD) is widely understood through the lens of Marsha Linehan’s biosocial theory, a foundational model that integrates biological and environmental factors. The theory proposes a transactional pathway to the disorder. It begins with a pre-existing biological vulnerability, such as heightened emotional sensitivity, intense reactivity, and a slow return to emotional baseline. This vulnerable individual is then raised within an "invalidating environment," one in which "the communication of private experiences, particularly emotions, is met with erratic, inappropriate, or dismissive responses from caregivers" [1,2].

Childhood adversity, including emotional abuse and neglect, is a primary and severe form of such invalidation. This chronic invalidation has a critical consequence: it fails to teach the child how to understand, label, tolerate, and modulate emotional arousal. As a result, the individual develops pervasive emotional dysregulation. This core deficit in emotion regulation, in turn, is believed to drive the behavioral dysregulation characteristic of BPD, such as impulsive acts, self-injury, and interpersonal turmoil, which are often desperate attempts to manage overwhelming and confusing emotional states [1,3,4].

The biosocial model is thus a process model, outlining a specific causal chain: "biological vulnerability and an invalidating environment transact to produce emotional dysregulation, which then leads to behavioral dysregulation" [5].

Mediation as a statistical test of theory

While correlational studies can establish that childhood adversity, emotional dysregulation, and impulsivity are all linked to BPD, they cannot illuminate the underlying process. Mediation analysis is a powerful statistical method that allows researchers to move beyond simple association to test a hypothesized psychological mechanism. It is designed to answer the question of how or why an independent variable (X) influences a dependent variable (Y). It does so by testing whether a third variable, the mediator (M), accounts for the relationship between X and Y [6].

In the context of the biosocial theory, mediation analysis provides a direct statistical test of the proposed causal pathway. It can formally examine whether the path from childhood adversity (X) to impulsive behavior (Y) is explained by the intervening role of emotional dysregulation (M). A significant mediation effect would provide strong empirical evidence that emotional dysregulation is the "bridge" that connects early adverse experiences to adult impulsive symptomatology [6].

Review of mechanistic models in BPD research

In recent years, a growing number of studies have employed mediation and moderation models to test these etiological pathways in BPD, providing increasing support for the biosocial framework. For instance, longitudinal studies have examined developmental models of BPD and non-suicidal self-injury (NSSI). Research has found that "emotion regulation difficulties (ERD) significantly mediated the association between childhood maltreatment and both NSSI and BPD symptoms, confirming that dysregulation is a key mechanism linking early adversity to later pathology" [7].

Similarly, Kenézlői et al. (2025), in a study comparing BPD and ADHD, found that childhood traumatization was associated with poorer adult personality functioning and that this effect was mediated more strongly by emotion dysregulation than by impulsivity itself. This highlights the primary role of emotional processes in translating trauma into functional impairment. Specifically, their mediation analysis revealed that "emotion regulatory capacity played a significant mediating role between childhood traumatization and the level of personality functioning" [8].

Schaich et al. (2021) provided an even more specific finding, demonstrating that the "impulse control difficulties" subcomponent of emotional dysregulation mediated the relationship between childhood emotional abuse and BPD symptoms. Their analysis revealed that "impulse control difficulties" was "the aspect of difficulties in emotion regulation that has the greatest impact on this association (B = 0.021, 95% CI: 0.003, 0.045)" [9]. These studies provide converging evidence for emotional dysregulation as a critical mediator. However, much of this research has been conducted in Western populations. Testing these mechanistic models in diverse cultural samples is essential to establishing the universality of the proposed pathway.

Rationale and hypothesis

The present study was designed to provide a rigorous test of a core tenet of the biosocial theory in an under-researched Indian clinical sample. By employing mediation analysis, we sought to move beyond describing the correlates of BPD to explaining the psychological mechanism that connects early experience to a key adult symptom. Specifically, we focused on negative urgency, the facet of impulsivity most strongly and consistently linked to BPD pathology [10].

Based on the biosocial model and the supporting literature, the following hypothesis was formulated: Total emotional dysregulation (as measured by the DERS-SF) will significantly mediate the positive relationship between total childhood adversity (as measured by the CECA-Q) and negative urgency (as measured by the UPPS-P (urgency, premeditation, perseverance, sensation seeking, and positive urgency)).

## Materials and methods

This cross-sectional study was conducted in Hyderabad, India, following ethical approval from the Institutional Ethics Committee of Malla Reddy Medical College for Women (Approval Number: MRMCWIEC/AP/81/2022). Participants were recruited through purposive sampling from outpatient psychiatry clinics affiliated with multiple teaching hospitals in Hyderabad. Consultant psychiatrists identified eligible patients during routine clinical assessments. The recruitment process involved initial screening by treating psychiatrists to identify potential participants who met DSM-5 (Diagnostic and Statistical Manual of Mental Disorders, 5th Edition) criteria for BPD, followed by referral to the research team for a detailed eligibility assessment. After a thorough explanation of the study procedures was provided, written informed consent was obtained from all participants. Diagnostic confirmation was then carried out, and eligible participants completed the study instruments.

Participants were included if they were between 18 and 45 years of age, had a primary diagnosis of BPD according to DSM-5 criteria confirmed by a structured clinical interview, were able to read and comprehend English or Hindi, were willing to provide written informed consent, and were clinically stable, defined as having no psychiatric hospitalization in the preceding four weeks and no acute suicidal crisis at the time of assessment. Exclusion criteria included active psychotic symptoms or a primary diagnosis of a schizophrenia spectrum disorder, current substance use disorder requiring detoxification, intellectual disability or cognitive impairment that precluded informed consent or reliable self-report, severe medical illness affecting cognitive function (such as uncontrolled epilepsy or severe traumatic brain injury), and inability to complete self-report questionnaires in English or Hindi.

The sample size was determined based on established guidelines for detecting indirect effects in mediation models. Using the bias-corrected bootstrap confidence interval method, the analysis was designed to achieve 80% power at a two-tailed significance level of α = 0.05. Drawing from previous studies on similar mediational pathways in BPD (Bertele et al., 2022 [7]; Schaich et al., 2021 [9]), medium effect sizes were anticipated for the path coefficients: Path a (Childhood Adversity → Emotional Dysregulation), r = 0.50, and Path b (Emotional Dysregulation → Negative Urgency), r = 0.50, with an expected indirect effect of approximately 0.25. Based on Fritz and MacKinnon (2007) [11] sample size guidelines, 34 participants were required to achieve 80% statistical power for medium effect sizes using the bias-corrected bootstrap method. To accommodate potential missing data and ensure adequate statistical power, the target sample size was increased to 45 participants, representing a 30% safety margin above the minimum required.

 Measures

Childhood Experience of Care and Abuse Questionnaire (CECA-Q)

The CECA-Q (Bifulco, Bernazzani, Moran, and Jacobs, 2005) [12] is a validated self-report instrument designed to retrospectively assess experiences of childhood neglect and abuse before age 17. The questionnaire was developed as a questionnaire version of the semi-structured Childhood Experience of Care and Abuse (CECA) interview and has demonstrated satisfactory reliability and validity as a self-report measure (Bifulco et al., 2005) [12]. The CECA-Q assesses multiple domains, including parental neglect (antipathy and neglect), physical abuse, and sexual abuse. For this study, the total adversity score was used, providing a comprehensive index of cumulative childhood adversity. The CECA-Q has been validated in multiple international samples and demonstrates good psychometric properties, with internal consistency coefficients ranging from 0.61 to 0.90 across subscales (Bifulco et al., 2005) [12].

Difficulties in Emotion Regulation Scale-Short Form (DERS-SF)

The DERS-SF is an 18-item short form of the original 36-item Difficulties in Emotion Regulation Scale (Gratz and Roemer, 2004). The DERS-SF maintains the six-factor structure of the original measure [13], assessing: (1) non-acceptance of emotional responses, (2) difficulties engaging in goal-directed behavior when distressed, (3) impulse control difficulties when upset, (4) lack of emotional awareness, (5) limited access to emotion regulation strategies, and (6) lack of emotional clarity. Each subscale contains three items rated on a five-point Likert scale (1 = almost never to 5 = almost always). The DERS-SF demonstrates excellent psychometric properties, with strong correlations (r = 0.91-0.98) with the full DERS, high internal consistency (α = 0.78-0.91), and comparable concurrent validity (Kaufman et al., 2016) [14]. The total score was used in this study as an index of overall emotional dysregulation.

Short UPPS-P Impulsive Behaviour Scale

The Short UPPS-P is a 20-item brief version of the 59-item UPPS-P Impulsive Behavior Scale, which assesses five distinct facets of impulsivity: (1) negative urgency, (2) positive urgency, (3) lack of premeditation, (4) lack of perseverance, and (5) sensation seeking. Each subscale contains four items rated on a four-point Likert scale (1 = agree strongly to 4 = disagree strongly). The negative urgency subscale, which assesses the tendency to act rashly when experiencing intense negative emotions, was the primary outcome variable of interest in this study. The Short UPPS-P demonstrates comparable psychometric properties to the full version, maintaining a similar factor structure, internal consistency (α > 0.80 for all subscales), and convergent validity while significantly reducing participant burden (Cyders et al., 2014) [14-16].

Permission to Use Scales

All scales used in this study are freely available for research purposes and do not require formal permission from copyright holders.

Data collection procedure

All participants completed the assessment battery in a private, quiet room at the outpatient clinic. Trained research assistants administered the questionnaires in a standardized order: (1) demographic information, (2) CECA-Q, (3) DERS-SF, and (4) Short UPPS-P. Participants were given the option to complete the questionnaires in English or Hindi based on their preference and comfort level. All questionnaires were presented in paper format. Research assistants remained available to answer questions and ensure comprehension but did not influence responses. The complete assessment required approximately 45-60 minutes.

Statistical analysis

All statistical analyses were conducted using IBM SPSS Statistics for Windows, Version 26 (Released 2018; IBM Corp., Armonk, New York). Descriptive statistics (means, standard deviations, and ranges) were calculated for all study variables. Prior to hypothesis testing, data were screened for normality, outliers, and missing values. No significant missing data were identified (less than 5% for any variable).

Preliminary analyses included (1) calculation of Pearson correlation coefficients to examine bivariate relationships among all study variables and (2) assessment of assumptions for mediation analysis (linearity, homoscedasticity, and absence of multicollinearity).

## Results

Participant characteristics

The final sample consisted of 45 adult patients diagnosed with BPD. The sample was predominantly young (73.3%, n=33 aged 18-27 years), female (93.3%, n=42), and highly educated, with 60% (n=27) having completed undergraduate degrees and an additional 13.3% (n=6) holding postgraduate qualifications. The majority of participants resided in urban areas (93.3%, n=42) and belonged to middle socioeconomic status households (93.3%, n=42). Regarding family structure, 73.3% (n=33) lived in nuclear families, while 26.7% (n=12) were from joint family systems. Complete demographic details are presented in Table 1.

**Table 1:** Sociodemographic Details

Variables	n	%
Age		
18-27 years	33	73.3
28-37 years	6	13.3
38-47 years	6	13.3
Gender		
Male	3	6.7
Female	42	93.3
Education		
Primary school (up to 6^th^ grade)	3	6.7
Secondary school (up to 10^th^ grade)	9	20
Graduate (degree)	27	60
Postgraduation	6	13.3
Residence		
Rural	3	6.7
Urban	42	93.3
Socio-economic status		
Low	3	6.7
Middle	42	93.3
Type of family		
Nuclear	33	73.3
Joint	12	26.7

Bivariate Correlations Among Key Variables

Before conducting the mediation analysis, it was necessary to establish that the prerequisite statistical conditions were met. This required demonstrating that the independent variable (Childhood Adversity), the proposed mediator (Emotional Dysregulation), and the dependent variable (Negative Urgency) were all significantly intercorrelated. Pearson correlation analyses were conducted to examine these relationships, as detailed in Table 2. The results confirmed that all variables were strongly and significantly associated in the hypothesized directions. Total Childhood Adversity was strongly correlated with both Total Emotional Dysregulation (r = 0.60, p < 0.001) and Negative Urgency (r = 0.55, p < 0.001). Furthermore, the mediator, Total Emotional Dysregulation, was very strongly correlated with the outcome, Negative Urgency (r = 0.72, p < 0.001). All three variables were also strongly correlated with overall BPD symptom severity. These significant bivariate relationships justify proceeding with the formal test of the mediation model [6].

**Table 2:** Pearson Correlation Matrix of Key Study Variables (N=45) ** p<0.001. All variables are total scores from their respective scales.

Variable	1. Adversity	2. Dysregulation	3. Negative Urgency	4. BPD Severity
1. Total Adversity (CECA-Q)	1			
2. Total Dysregulation (DERS-SF)	.60**	1		
3. Negative Urgency (UPPS-P)	.55**	.72**	1	
4. BPD Severity (BSL-23)	.58**	.65**	.61**	1

The Mediation Model

To test the central hypothesis that emotional dysregulation mediates the link between childhood adversity and negative urgency, a mediation analysis was performed using the Hayes PROCESS macro (Model 4) for SPSS with 5,000 bootstrap samples. Total Childhood Adversity was entered as the independent variable (X), Total Emotional Dysregulation as the mediator (M), and Negative Urgency as the dependent variable (Y) [6].

The results of the mediation analysis, presented in Table 3, provide strong support for the hypothesis. The analysis first confirmed a significant total effect of childhood adversity on negative urgency (c-path: B = 0.39, p < 0.001), indicating that greater adversity was associated with higher negative urgency. The path from childhood adversity to the mediator, emotional dysregulation, was also highly significant (a-path: B = 0.75, p < 0.001), as was the path from the mediator to negative urgency, controlling for adversity (b-path: B = 0.41, p < 0.001).

**Table 3:** Mediation Analysis of Emotional Dysregulation in the Relationship Between Childhood Adversity and Negative Urgency (N=45)

Path	Coefficient (B)	Std. Error	t-value	p-value	95% Confidence Interval
Total Effect (c-path)	0.39	0.09	4.33	.001	[0.21, 0.57]
Adversity -> Dysregulation (a-path)	0.75	0.15	5.00	.001	[0.45, 1.05]
Dysregulation -> Urgency (b-path)	0.41	0.08	5.13	.001	[0.25, 0.57]
Direct Effect (c'-path)	0.08	0.11	0.76	0.450	[-0.14, 0.30]
Indirect Effect (a*b)	0.31	0.09			[0.14, 0.52]

The crucial test of mediation is the indirect effect. The bootstrapped analysis revealed a significant indirect effect of childhood adversity on negative urgency through emotional dysregulation, with a point estimate of 0.31. The 95% confidence interval for this indirect effect did not contain zero (0.14, 0.52), confirming that emotional dysregulation is a statistically significant mediator [6].

Finally, after accounting for this indirect effect, the direct effect of childhood adversity on negative urgency (c'-path) was no longer statistically significant (B = 0.08, p = 0.450). This pattern, in which a previously significant total effect becomes non-significant after inclusion of the mediator, is consistent with full mediation. This finding suggests that the influence of childhood adversity on the tendency to act impulsively under negative affect is largely explained by its detrimental impact on emotion regulation capacity [6].

## Discussion

Confirmation of the biosocial pathway in an Indian context

The primary finding of this investigation is the strong empirical support it provides for a core pathway of Linehan’s biosocial theory of BPD. By demonstrating that emotional dysregulation fully mediates the relationship between childhood adversity and negative urgency, this study moves beyond mere correlation to illuminate a key psychological mechanism. The results show how early adverse experiences can translate into a hallmark feature of adult BPD pathology.

In this Indian clinical sample, as predicted by the theory, childhood adversity appears to foster deficits in emotion regulation, and it is these deficits that, in turn, give rise to the tendency to act impulsively and rashly when experiencing distressing emotions. This finding serves as an important cross-cultural validation of the biosocial model, suggesting that this fundamental etiological pathway is robust and applicable beyond the Western contexts in which it was originally developed and primarily tested [1].

Emotional dysregulation as the active ingredient of trauma’s impact

The statistical finding of full mediation carries profound psychological and clinical implications. The fact that the direct path from childhood adversity to negative urgency became non-significant after accounting for emotional dysregulation suggests that adversity, on its own, may not have a direct, unmediated link to this specific form of impulsivity. Instead, its primary pathogenic effect appears to be the damage it inflicts upon the psychological “machinery” of emotion regulation.

This allows for a more refined understanding of the mechanism. It is the disrupted machinery (the acquired deficits in emotional awareness, clarity, acceptance, and strategic control) that directly precipitates impulsive action in the face of distress. The memory of the trauma itself (the independent variable) initiates the pathological process, but it is the resulting state of emotional dysregulation (the mediator) that serves as the “active ingredient” directly driving the problematic behavior (the dependent variable).

This mechanistic understanding provides a powerful and parsimonious explanation for a central feature of BPD. It clarifies that negative urgency is not simply a “bad habit” or a character flaw, but a predictable consequence of having never developed the necessary internal skills to tolerate and manage intense affective states, a failure directly attributable to the invalidating environment of childhood adversity [1].

Comparison with relevant literature

The present findings are broadly consistent with, and in some respects stronger than, other recent mechanistic studies of BPD. For example, longitudinal studies have also found significant mediation effects of ERD in the link between childhood maltreatment and BPD symptoms. However, some models have demonstrated partial mediation, meaning that a significant direct effect of maltreatment on BPD remained even after accounting for ERD. The finding of full mediation in the present study for the specific pathway to negative urgency is a stronger result, suggesting that for this core component of impulsivity, emotional dysregulation is a uniquely powerful explanatory variable [7].

The results also align with the conclusions of Kenézlői et al. (2025), who found that the impact of childhood trauma on overall personality functioning was mediated more strongly by emotion dysregulation than by impulsivity, again pointing to the primacy of the emotional deficit. Furthermore, the work of Schaich et al. (2021) helps to specify which aspect of the mediator may be most active; they found that "impulse control difficulties" in particular mediated the link between emotional abuse and BPD symptoms. This suggests that the "Impulse" subscale of the DERS is likely a key component of the overall mediation effect captured by the DERS total score in the present analysis [8,9].

In contrast to more complex models that have tested moderation effects, for example, unsupported hypotheses that trait impulsivity would moderate the trauma-dysregulation link, the present study demonstrates that a clear and direct mediation model provides a robust explanation for the data [7].

Clinical and public health implications

Confirmation of this mediational pathway has direct and important implications for both the treatment and prevention of BPD.

First, from a clinical treatment perspective, this finding provides a strong, evidence-based rationale for prioritizing skills-based psychotherapies that directly target the mediator variable, emotional dysregulation. If the path from trauma to impulsivity operates through deficits in emotion regulation, then the most effective point of intervention is to address those deficits. This empirically validates the core structure of Dialectical Behavior Therapy (DBT), whose central modules, "Mindfulness, Distress Tolerance, Emotion Regulation, and Interpersonal Effectiveness," are designed to directly target components of emotional dysregulation [1].

The results support a strategic shift in clinical focus, moving beyond purely narrative-based trauma processing toward skills-based interventions that explicitly build the capacity for emotional management. This underscores the need for training, dissemination, and cultural adaptation of therapies such as DBT within the Indian mental healthcare system.

Second, from a public health and prevention perspective, the model points toward a clear strategy. If emotional dysregulation is the critical link between high-risk environments and later pathology, then interventions that strengthen emotion regulation skills in childhood may serve as effective preventive measures. Universal or targeted programs delivered in schools and communities that teach emotional literacy, coping skills, and distress tolerance to children, particularly those at high risk due to adverse home environments, could potentially disrupt this pathological pathway before BPD symptoms consolidate in adolescence and adulthood.

Strengths and limitations

The primary strength of this study is its use of a rigorous, bootstrapping-based mediation analysis to test a specific, theory-driven psychological mechanism. This approach moves the research beyond simple description to an explanation of process, providing findings with stronger theoretical and clinical utility.

However, the study’s limitations must be carefully considered. The most significant limitation is the cross-sectional design. While the tested mediation model is theoretically coherent and widely accepted (i.e., adversity precedes dysregulation, which precedes impulsive acts), the single-timepoint data cannot prove this temporal sequence or establish causality. A longitudinal study, tracking individuals over time, would be required to definitively confirm the proposed causal chain.

As detailed in the participant characteristics, the sample was predominantly young (73.3% aged 18-27 years), female (93.3%), urban (93.3%), and well educated (73.3% graduates or above). This small and demographically homogeneous sample (young, urban, educated females) is another major limitation that restricts the generalizability of the findings to other populations in India. Finally, the reliance on self-report measures carries the risk of shared method variance, which may have inflated the observed correlations to some degree.

## Conclusions

By demonstrating that emotional dysregulation fully mediates the relationship between childhood adversity and negative urgency, this study provides powerful, mechanism-based support for the biosocial theory of BPD within an under-researched Indian cultural context. This finding illuminates the critical pathway through which early traumatic experiences exert their pathogenic influence on adult behavior, clarifying that their impact is channeled through disruption of emotion regulation capacities.

These results have significant clinical resonance, providing a clear, empirically grounded mandate for prioritizing emotion regulation-focused interventions, such as Dialectical Behavior Therapy, in the treatment and prevention of BPD in India and globally. The findings underscore the importance of addressing the mechanistic core of the disorder rather than merely its surface manifestations, offering promise for more targeted and effective therapeutic approaches.
